# Biofilm adaptation and mucosal immune dysregulation in recalcitrant chronic rhinosinusitis: from pathogenesis to a therapeutic roadmap

**DOI:** 10.3389/fimmu.2026.1797096

**Published:** 2026-04-14

**Authors:** Shiwang Tan, Ju Lai, Shaoqing Yu

**Affiliations:** Department of Otolaryngology, Head & Neck Surgery, Tongji Hospital, School of Medicine, Tongji University, Shanghai, China

**Keywords:** biofilm, chronic rhinosinusitis, interspecies interaction, novel therapeutics, staphylococcus aureus

## Abstract

The management of chronic rhinosinusitis (CRS) is frequently complicated by treatment recalcitrance, a phenomenon primarily driven by the persistence of microbial biofilms. Beyond their traditional role as a physical barrier against antibiotics, recent evidence positions biofilms as sophisticated immune modulators that actively perpetuate mucosal dysbiosis. This review synthesizes the pathological continuum of biofilm-associated CRS, elucidating how biofilm derived pathogen associated molecular patterns (PAMPs) trigger the release of epithelial alarmins (TSLP, IL-33, IL-25), thereby fueling a maladaptive Type 2 inflammatory loop. We further examine bacterial survival strategies, such as the formation of small colony variants (SCVs) and intracellular “Trojan Horse” reservoirs, which render conventional functional endoscopic sinus surgery (FESS) and antimicrobial monotherapies insufficient for complete eradication. Crucially, we discuss the current diagnostic disconnect where standard cultures fail to detect biofilm burdens. Finally, we propose a therapeutic paradigm shift from a purely bactericidal approach to one of ecological restoration. By integrating cutting-edge strategies, including matrix-degrading enzymes, bacteriophage cocktails, and Nasal Microbiota Transplantation (NMT), we construct a multi-dimensional framework aiming to restore sinonasal homeostasis. Together, these emerging strategies support a shift from pathogen suppression alone toward ecological and immunologic rebalancing of the sinonasal mucosa, offering a more durable conceptual framework for overcoming treatment recalcitrance.

## Introduction

Chronic rhinosinusitis (CRS) is a common disease in rhinology characterized by persistent inflammation of the nasal and sinus mucosa, affecting approximately 10% of the population (1) and imposing a heavy burden on patients’ quality of life and the healthcare system. Recalcitrant CRS refers to persistent disease that remains partly controlled or uncontrolled despite appropriate standard medical or surgical treatment, with ongoing symptom burden, objective inflammatory findings, or repeated need for rescue therapy (2). Over the years, extensive research has been conducted on its pathogenesis, with current theories implicating factors such as superantigens, osteitis, eosinophilic infiltration, and ciliary dysfunction. However, these theories do not fully explain the resistance to conventional therapy observed in some patients, particularly those with recalcitrant CRS.

Over the past decade, the microbial biofilm (MBF) hypothesis has gained recognition as a central mechanism in the pathophysiology of recalcitrant CRS (3, 4). Although biofilms have been detected in a subset of healthy controls, most comparative studies report a substantially higher prevalence in CRS than in non-CRS mucosa, suggesting that biofilms are better interpreted as contributors to disease persistence rather than universal features of healthy sinonasal mucosa. The detection rate of biofilms in CRS is as high as 75% (5). Biofilms act as a persistent reservoir for pathogens (6, 7), leading to chronic inflammation and symptom exacerbation (8). However, there is currently no unified standard for the detection and treatment of biofilm-related CRS, and this area remains under exploration.

This narrative review synthesizes literature from PubMed, Embase, and Web of Science published between January 2000 and December 2025. Search strategies combined keywords such as “chronic rhinosinusitis,” “biofilm,” “*Staphylococcus aureus*,” “interspecies interaction,” and “novel therapeutics.” We prioritized randomized controlled trials, systematic reviews, and mechanistic studies (animal/ex vivo) that offer insights into biofilm pathophysiology or clinical management. Non-English articles and studies focusing solely on planktonic bacteria were excluded. The final selection aims to construct a comprehensive framework spanning from molecular mechanisms to clinical translation.

This review aims to systematically organize the existing literature, starting from the definition and theoretical basis of microbial biofilms, to elaborate on their microbial composition in CRS, their complex immune interactions with the host, and their clinical relevance, while also analyzing current diagnostic challenges and therapeutic frontiers. By integrating fragmented research, we aim to construct a complete knowledge framework from molecular mechanisms to clinical applications. This review not only seeks to integrate the complete pathological chain “from the initiation at the epithelial barrier to downstream immune effects and dysbiosis” but also to explore a new therapeutic paradigm “based on restoring host-microbe homeostasis,” ultimately providing new insights for improving the clinical prognosis of patients with recalcitrant CRS.

## General characteristics of microbial biofilms

A microbial biofilm is a highly structured community of microorganisms encased within a self-produced extracellular polymeric substance (EPS) matrix, which adheres to biological or non-biological surfaces. This structure not only serves as a microbial habitat but also forms a physical barrier through the EPS matrix, enhancing persistence in various environments (9). Its key biological properties make it a perfect fortress for chronic infections. The physical barrier of the EPS matrix, the low metabolic activity of the bacteria within, and the presence of “persister cells” render biofilms up to 1000 times more tolerant to antibiotics than their planktonic counterparts (10, 11). Furthermore, the EPS forms a physical and chemical barrier that not only blocks antibiotics but also effectively helps microorganisms evade the host’s immune system, including resisting phagocytosis by phagocytes and neutralization by antibodies (11). This protective mechanism is a key factor enabling biofilms to persist in the host, leading to chronic infections.

## Pathophysiology of chronic rhinosinusitis

The core pathological features of CRS are persistent inflammation, impaired mucosal barrier function, and mucociliary clearance dysfunction. In recent years, the concept of CRS endotypes has been widely accepted (12), primarily dividing CRS into Type 2 inflammation (characterized by eosinophilic infiltration and cytokines such as IL-5) and non-Type 2 inflammation (characterized by neutrophilic infiltration and TH1/TH17 pathways), based on the key immune pathways driving the inflammation (13).This theoretical framework provides crucial support for understanding how microbial biofilms induce heterogeneous immune responses.

## Biofilm formation and molecular adaptation

### Core pathogens and microbial ecology

Microbial biofilms have been confirmed to exist in various infections, including those related to implants, the gut, the upper respiratory tract (including pharyngitis and sinusitis), and even colorectal cancer (14).Both bacteria and fungi can form biofilms, and their ability to do so is closely related to the microbial species and the surrounding environment (15, 16).The microbial biofilm in CRS is not composed of a single pathogen but is a complex micro-ecosystem. *Staphylococcus aureus* and Pseudomonas aeruginosa are the most frequently detected key bacterial builders and are closely associated with disease severity (8, 17, 18). Additionally, studies have found that some less common bacteria may also play important roles.

### Bacterial molecular regulation and adaptive persistence

Biofilm development and persistence are strictly regulated by molecular switches. At the regulatory level, the accessory gene regulator (agr) quorum-sensing system acts as a molecular switch that is essential for initiating biofilm detachment (19). This agr-dependent process orchestrates the release of phenol-soluble modulins (PSMs), surfactant-like peptides that function as molecular scissors to degrade the matrix, thereby driving the active dispersal of bacteria to seed new infection sites (20, 21). Unlike planktonic bacteria, biofilm-embedded pathogens in CRS employ sophisticated metabolic reprogramming to evade eradication. Recent transcriptomic analyses reveal that oxygen depletion within the biofilm matrix triggers a metabolic switch to anaerobiosis, inducing a dormant “persister” state that renders metabolic-dependent antibiotics ineffective. Specifically, *Staphylococcus aureus* often transitions into SCVs within the sinonasal mucosa. As recently characterized by An et al. (22) in clinical isolates, these SCVs are distinguished by specific defects in the electron transport chain, leading to a reduced membrane potential that effectively blocks the uptake of cationic antibiotics like aminoglycosides. This “metabolic shielding,” rather than just the physical EPS barrier, is increasingly recognized as a primary driver of treatment recalcitrance.

Beyond the protection of the extracellular matrix, S. aureus biofilms exhibit sophisticated survival strategies characterized by intracellular persistence and phenotypic plasticity. Using ex vivo confocal microscopy on clinical biopsies, Tan et al. identified intracellular S. aureus reservoirs in 39% of CRS patients, proposing a “Trojan Horse” mechanism where bacteria evade host immunity and topical antibiotics within epithelial cells, subsequently reseeding the mucosa to cause recurrence (23). Furthermore, under the selective pressure of the inflammatory microenvironment, S. aureus can undergo phenotypic switching into SCVs. These electron-transport-defective variants exhibit slow growth and reduced virulence factor expression but enhanced intracellular survival and antibiotic tolerance (24). This reversible phenotype switching allows the pathogen to persist in a dormant state during treatment and resuscitate when conditions are favorable, acting as a core driver of recalcitrance. More specifically, CRS associated isolates exhibit distinct evolutionary adaptations. Tuchscherr et al. discovered that these strains undergo metabolic reprogramming, characterized by reduced glycolysis and cytotoxicity. This “stealth mode” allows the pathogen to persist in the nutrient-limited sinus mucosa without provoking acute host clearance (25). Furthermore, Nepal et al. demonstrated that S. aureus from chronic rhinosinusitis with nasal polyps (CRSwNP) patients is significantly enriched with prophages encoding the Human Immune Evasion Cluster (IEC). Key genes such as scn (Staphylococcal complement inhibitor) directly block complement activation, effectively paralyzing the recruitment of neutrophils despite the high inflammatory burden (26).

### Emerging evidence for inter-kingdom interactions

Emerging evidence suggests that bacteria and fungi may engage in synergistic interactions within polymicrobial communities in CRS, although direct evidence for polymicrobial biofilm architecture remains limited by current detection methods. Boase et al. (27) provided mechanistic support in a sheep model, showing that fungal spores alone failed to establish biofilms because of effective mucociliary clearance, whereas co-inoculation with S. aureus created a permissive inflammatory niche that enabled fungal adherence and biofilm establishment. Furthermore, metagenomic advances have expanded this landscape beyond S. aureus. Dellière et al. (28) identified a high-abundance co-occurrence of Haemophilus influenzae and Aspergillus fumigatus in fungal ball rhinosinusitis. This co-occurrence pattern raises the possibility that H. influenzae may facilitate fungal persistence through mechanisms potentially involving mucociliary inhibition, as observed *in vitro* for Haemophilus species. Collectively, these findings support the possibility of clinically relevant inter-kingdom interactions, but their prevalence, spatial organization, and pathogenic significance in CRS remain incompletely defined.

## Host immune response at the biofilm interface

### Genetic susceptibility: the starting point

The host’s genetic background plays a significant role in CRS susceptibility, with the functional polymorphism of the T2R38 bitter taste receptor serving as a well-studied example. This receptor is expressed on the ciliated epithelial cells of the sinonasal cavity and can recognize quorum-sensing molecules, such as acyl-homoserine lactones produced by gram-negative bacteria, triggering a calcium-dependent increase in nitric oxide (NO) production that enhances innate immune defense by promoting mucociliary clearance and direct bactericidal effects (29).Studies have confirmed that individuals carrying the non-functional AVI allele exhibit blunted NO and ciliary responses, which are associated with weaker innate immune function, making them more susceptible to gram-negative bacterial infections and resulting in a significantly higher detection rate of *in vivo* biofilms directly observed on sinonasal mucosa (5).This supports a potential pathogenic sequence wherein specific host genotypes contribute to innate immune deficiencies, thereby facilitating bacterial colonization and subsequent biofilm formation *in vivo*, highlighting TAS2R38 polymorphisms as promising genetic markers for individualized risk assessment in CRS.

### Epithelial-driven type 2 inflammation axis

**Figure 1:**
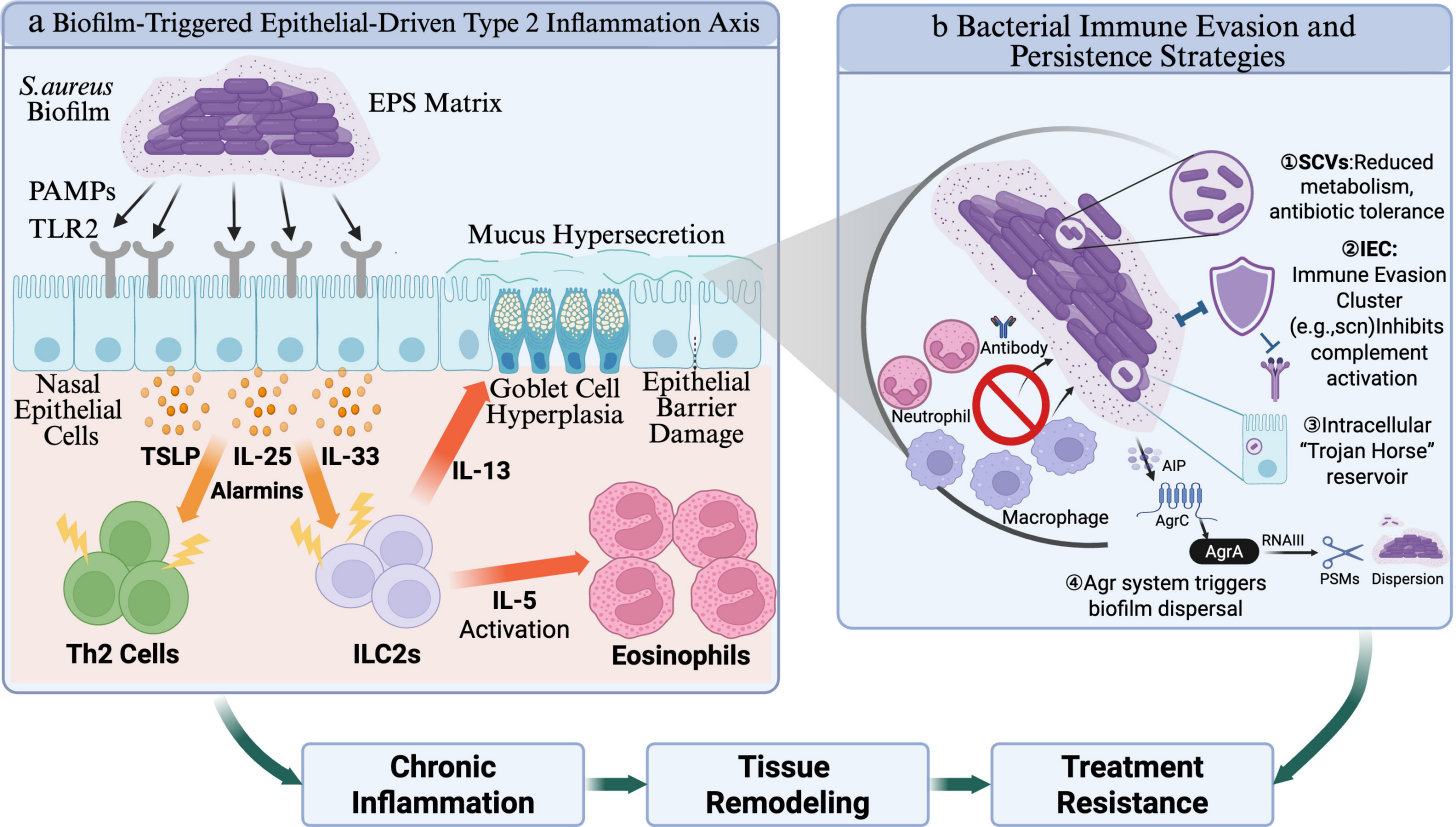
Microbial biofilm-driven immunopathological pathways in chronic rhinosinusitis. **(a)** Biofilm-Triggered Epithelial-Driven Type 2 Inflammation Axis: S. aureus biofilms stimulate nasal epithelial cells via PAMP–TLR2 signaling, triggering the release of epithelial alarmins (TSLP, IL-33, and IL-25). These cytokines activate ILC2s and Th2 cells, driving IL-5- and IL-13-dominant responses that recruit eosinophils and promote goblet cell hyperplasia, mucus hypersecretion, epithelial barrier dysfunction, and polypoid remodeling. **(b)** Bacterial Immune Evasion and Persistence Strategies: Despite ongoing host inflammatory responses, biofilm-associated bacteria persist through four core mechanisms: (1) Metabolic Stealth, in which bacteria undergo metabolic reprogramming and switch to SCVs, reducing susceptibility to antibiotics and immune clearance; (2) Immune Evasion, in which prophage-encoded IEC proteins inhibit complement activation and impair neutrophil recruitment and killing; (3) Intracellular “Trojan Horse” Reservoirs, in which bacteria invade epithelial cells and establish protected intracellular niches, thereby evading topical therapy and host immune surveillance and later reseeding the mucosa to promote recurrence; and (4) Active Dispersal, in which the agr quorum-sensing system induces PSMs and proteases that degrade the biofilm matrix, allowing bacterial clusters to disperse and colonize new mucosal sites. Together, these host-pathogen interactions create a self-reinforcing cycle of chronic inflammation, tissue remodeling, and treatment recalcitrance in CRS. Created in BioRender. Tan, S. (2026) https://BioRender.com/gg8rc84.

The core mechanism behind the superantigen-independent induction of a TH2 type skew by S. aureus biofilms lies in their ability to directly activate epithelial cells, the first line of innate immunity (30). Sinus mucosal epithelial cells are not just a physical barrier but also “sentinels” of the immune system. S. aureus and its biofilms can interact with Toll-like receptor 2 (TLR2) on the surface of epithelial cells via their pathogen-associated molecular patterns (PAMPs). This initial contact triggers the rapid release of a key set of initial signaling molecules from the epithelial cells, known as “alarmins,” primarily including thymic stromal lymphopoietin (TSLP), interleukin-33 (IL-33), and interleukin-25 (IL-25). These alarmins are the core upstream cytokines that drive type 2 inflammation, potently activating type 2 innate lymphoid cells (ILC2s) and Th2 helper T cells in the submucosa (31–33). These activated cells are the main source of the downstream key effector cytokines IL-5 and IL-13. IL-5 drives the recruitment and activation of eosinophils (34), while IL-13 promotes goblet cell hyperplasia, mucus hypersecretion, and impaired epithelial barrier function (35), these are the core pathological features of CRSwNP (**Figure 1**).

### Spectrum of immune responses

The host’s immune response to microbial biofilms presents a highly complex inflammatory phenotype, as evidenced by diverse clinical research findings. Studies have demonstrated that in patients not receiving steroid therapy, biofilm presence is significantly associated with a local TH1-type inflammatory response characterized by elevated interferon-gamma levels and neutrophilic infiltration. Particularly noteworthy is the observation that systemic steroid treatment can significantly attenuate this TH1 inflammatory response, providing important clues for interpreting the divergent immune responses reported across different studies (36). From a mechanistic perspective, the association between *Staphylococcus aureus* biofilms and TH2-type immune responses has been well established (37).This TH2-skewed response manifests as significantly elevated levels of interleukin-5 and eosinophil cationic protein, independent of bacterial superantigen activity. Histological studies further corroborate these findings, demonstrating significantly enhanced expression of plasma cell marker CD27 and eosinophil major basic protein in the nasal mucosa of biofilm-positive patients (38), thereby providing solid morphological evidence for TH2-type inflammatory responses. These findings carry important implications for clinical treatment strategies. Different biofilm types may trigger specific immune backgrounds, necessitating personalized treatment approaches based on individual characteristics. Particularly for patients with *Staphylococcus aureus* biofilm-associated conditions showing TH2 skewing, targeted therapies addressing specific inflammatory pathways may offer more precise treatment options. Concurrently, healthcare providers need to recognize that steroid treatments may alter the immune response characteristics associated with biofilms, which has significant implications for accurate disease mechanism interpretation and treatment outcome assessment.

### Intracellular recognition and persistent inflammation

Even more noteworthy, research has found that the pathogenic mechanism of microbial biofilms extends from the mucosal surface to the cell interior. S. aureus biofilms can activate intracellular pattern recognition receptors (PRRs), such as Nod2 and its downstream NF-κB signaling pathway (39).In CRS patients with nasal polyps and S. aureus biofilms, the expression of the AIM2 inflammasome is also significantly upregulated (40).Since Nod2 and AIM2 recognize intracellular bacterial peptidoglycan degradation products and double-stranded DNA, respectively, their activation strongly suggests that bacterial components are entering the host cells. These complex signaling pathways ultimately translate into specific histopathological changes, including a direct association with eosinophil and plasma cell infiltration, as well as the induction of epithelial cell apoptosis, thereby disrupting the mucosal barrier (41).While superantigens are known triggers of Type 2 inflammation, S. aureus biofilms possess intrinsic immunomodulatory properties independent of superantigen activity. Foreman et al. demonstrated that biofilm-positive CRS tissues exhibit a distinct T-helper 2 (Th2) skew characterized by elevated IL-5 and ECP, even in the absence of demonstrable superantigens (37).

However, the persistence of biofilms despite this robust inflammatory response implies the existence of sophisticated bacterial evasion strategies. Recent molecular evidence reveals that S. aureus employs multi-layered mechanisms to survive this hostile environment.

Thus, a multi-dimensional pathogenic axis is established: On the host side, biofilms trigger an epithelial-driven “PAMPs-TLR2-Alarmins” cascade that fuels maladaptive type 2 inflammation; on the pathogen side, S. aureus utilizes prophage-encoded immune evasion clusters and metabolic downregulation to actively withstand this attack. This dual mechanism, host immune hijacking coupled with bacterial molecular stealth, comprehensively explains the recalcitrance of biofilm-associated CRS and provides a solid theoretical basis for developing targeted therapies that disrupt both the inflammatory loop and bacterial persistence (**Figure 1**).

Despite this robust intracellular and extracellular immune activation, the bacterial evasion mechanisms described in Section 4.2 allow the biofilm to withstand these attacks, leading to the chronic recalcitrance observed clinically.

## Clinical reality: diagnosis, phenotypes, and systemic impact

### Biofilms in the united airway context

The concept of ‘United Airway Disease’ posits that the upper and lower respiratory tracts function as an integrated unit. However, evidence relevant to this framework should be interpreted across different biological scales. Hernaiz-Leonardo et al. demonstrated that in CRS patients, the sinus and lung microbiomes exhibit significant dissimilarity in diversity and composition, with no evidence of clustering between paired samples, arguing against simple bulk bacterial flow via microaspiration as the dominant explanation for lower-airway microbial patterns (42). At the same time, these community-level findings do not exclude the possibility that selected bacterial strains may still be shared across anatomical sites. This distinction is important when considering cystic fibrosis studies, in which genetically identical P. aeruginosa clones have been shown to persist in the sinuses and re-infect the lungs (43, 44).Crucially, Armbruster et al. showed that in such contexts, persistence is driven by ‘genomic erosion’ within the sinus niche, where pathogens adapt before migrating (45). Thus, in CRS, the sinus may function predominantly as an independent inflammatory niche, although limited available data do not completely exclude selective strain sharing between airway compartments.

Furthermore, biofilm prevalence appears to be endotype-dependent and closely linked to epithelial barrier dysfunction (46). In patients with CRSwNP, biofilms are strongly associated with a Type 2 inflammatory signature. Sun et al. reported that biofilm-positive CRSwNP patients in the Chinese population exhibited significantly elevated serum total IgE levels and higher disease severity scores compared to biofilm-negative counterparts (47). Mechanistically, factors secreted by S. aureus biofilms (SABSFs) have been shown to directly drive hallmark features of Type 2 inflammation. Houtak et al. demonstrated in a rat model that exposure to SABSFs significantly induced mast cell infiltration, goblet cell hyperplasia, and mucosal damage, which was more pronounced with strains isolated from CRSwNP patients compared to controls (33). Collectively, these findings underscore that biofilms are not merely local irritants but are integral drivers of the systemic inflammatory burden in specific CRS endotypes.

### Clinical significance, genomic adaptations, and prognosis

The clinical intractability of CRSwNP is mirrored by specific genomic adaptations. Goldie et al. (48)demonstrated that S. aureus isolates from CRSwNP patients are genetically distinct from those in controls, often exhibiting a loss of core virulence genes (e.g., icaC, scn, hlgA). The loss of icaC, paradoxically, is associated with a shift towards persistent biofilm formation and immune evasion rather than acute virulence. Moreover, these biofilm-forming strains are strongly linked to the “United Airway” pathology. CRSwNP patients colonized by S. aureus show a significantly higher prevalence of comorbid asthma (55.5%) compared to controls (13.3%). This systemic link is likely driven by the enterotoxin gene cluster (egc) found in these strains, which acts as a superantigen depot to sustain Type 2 inflammation across the entire respiratory tract. Additionally, the identification of invasive virulence factors (lukE/D, splA) explains the failure of topical therapies, as these factors facilitate intracellular bacterial survival, creating reservoirs inaccessible to saline irrigation. Numerous clinical studies consistently show that the presence of microbial biofilms is associated with more severe clinical manifestations of CRS and a poorer prognosis. Biofilm-positive patients typically exhibit more severe radiological changes and more significant endoscopic signs of inflammation before surgery. Postoperatively, biofilm-positive patients have a significantly prolonged mucosal healing time (49), and their long-term improvement in quality of life is also significantly lower than that of biofilm-negative patients (50).

### Diagnostic challenges and technology comparison

One of the core challenges in CRS diagnosis today is the diagnostic disconnect: there is a huge discrepancy between conventional clinical detection methods and the actual pathophysiological state of the mucosa. The most commonly used clinical method, nasal swab culture, is highly insensitive to biofilm bacteria deeply embedded in the matrix because it primarily detects planktonic bacteria, leading to results that severely misrepresent the true pathogen spectrum (51, 52). To address this deficiency, researchers have developed various techniques based on biopsy and advanced imaging, including scanning electron microscopy (SEM), confocal laser scanning microscopy (CLSM) combined with BacLight, and fluorescence *in situ* hybridization (FISH) (**Table 1**).

**Table 1:** Representative studies of biofilm detection methods and associated findings in CRS.

References	Method	Bacteria	Materials	Sample size	Outcomes	Model	Date
Wu X et al. (53)	H&E/SEM	*Staphylococcus aureus*	rabbit	18	IL-1β,IL-8,and TNF-α↑;IL-4 and IL-5↓	CRS	2018
Marcinkiewicz et al. (54)	SEM	*Staphylococcus epidermidis*	human	10	MPO↓	CRS	2015
Hekiert et al. (36)	SEM	/	Human	60	INF-γ, G-CSF, and MIP-1β↑	CRS	2009
A. Foreman et al. (37)	FISH/CSLM	*Staphylococcus aureus*	Human	68	IL-5,IL-6 and ECP↑	CRS	2011
Arjomandi et al. (38)	H&E/FISH	S.aureus, H. influenzae, *P. aeruginosa*, and nonspecific fungi	Human	29	CD27,EMBP↑	CRS	2013
Jardeleza et al. (40)	FISH	*Staphylococcus aureus*	Human	16	IFN-γ,IL-1β↑	CRSwNP	2013
Cantero et al. (39)	CSLM	*Staphylococcus aureus*	Human	4	IL-6,CXCL1, CXCL2↑	sinonasal explant cultures	2013
Alkis J Psaltis et al. (55)	CSLM/SEM	/	Human	62	Lactoferrin ↓	CRS	2008

↑ and ↓ indicate relatively increased or decreased levels compared with the non-biofilm or comparator group defined in each original study.

For instance, the electron beam and vacuum environment used in SEM alone may damage some live bacteria and can only observe a 3D structure resembling a biofilm, which can easily lead to false positives. Some research suggests that BacLight/CSLM and FISH/CSLM are complementary techniques for biofilm detection. Although their identification results for the same sample may not be completely consistent, they are suitable for different types of research, BacLight/CSLM is suitable for comparing biofilm-positive and biofilm-negative CRS patients, while FISH/CSLM can be used to identify specific bacterial and fungal species (56).However, as detailed in **Table 2**, these methods vary significantly in terms of cost-effectiveness and clinical feasibility. While SEM remains the research gold standard, its high cost and complex sample preparation limit its routine clinical use compared to molecular methods like PCR. Despite the precision of advanced imaging techniques, a substantial gap remains between research methodology and routine clinical practice. In outpatient settings, the diagnosis of biofilm-associated CRS remains largely presumptive, often relying on surrogate clinical features such as persistent purulence despite adequate surgery, poor response to appropriate therapy, or early postoperative recurrence. A major unmet need in the field is the development of clinically feasible diagnostic tools that can identify biofilm-associated disease in real time or near real time. Future point-of-care approaches may target biofilm specific extracellular matrix components, such as extracellular DNA or polysaccharide-rich matrix signatures, but technological innovation alone will not be sufficient. Equally important is the establishment of standardized identification criteria and harmonized workflows, including biopsy location, sample processing, imaging thresholds, and species-identification strategies. Such standardization will be essential to distinguish true adherent biofilm from mucus, debris, or transient colonization, and to improve comparability across studies, patient stratification, and the design of therapeutic trials.

**Table 2:** Comparison of biofilm detection technologies.

Technology name	Principle	Advantages	Disadvantages	Relative cost	Clinical feasibility
Scanning Electron Microscopy (SEM)	Electron beam scans surface for high-res 3D images.	Gold standard for visualization; provides direct evidence of biofilm structure.	Complex sample preparation (dehydration, fixation), risk of creating artifacts, cannot identify species.	High (Requires specialized EM facility and maintenance)	Low (Labor-intensive; turnaround time >24h; not suitable for routine screening)
Confocal Laser Scanning Microscopy (CSLM)	Laser scanning for optical sectioning and 3D reconstruction.	Can image the 3D structure of living biofilms; combined with fluorescent probes, can distinguish between dead/live cells.	Relatively limited field of view, limited penetration of the matrix, expensive equipment.	High	Low to Moderate (Requires immediate processing; limited to specialized centers)
Peptide Nucleic Acid Fluorescence *In Situ* Hybridization (PNA-FISH)	Peptide nucleic acid probes bind to specific bacterial rRNA.	Allows for rapid, specific identification of species *in situ* within the biofilm.	Probes need to be pre-designed, relatively high cost, limited number of species can be detected at one time.	Moderate to High	Moderate (Requires fluorescence microscope)
Crystal Violet Staining	Dye binds to negatively charged biofilm components.	Simple, fast, inexpensive, suitable for high-throughput screening.	Cannot distinguish between dead/live cells, provides no structural information, poor specificity.	Low	High (Technically simple, but limited clinical utility due to low specificity)
PCR-based methods	Detects biofilm-associated genes (e.g.,icaA/D) or bacterial load.	Extremely high sensitivity, can detect very small sample quantities.	The presence of a gene does not guarantee biofilm formation, provides no structural information.	Moderate	High (Standardized workflows available; rapid turnaround;widely accessible)

## New therapeutic paradigms

### Limitations of standard therapies

Before exploring novel strategies, it is crucial to delineate the boundaries of standard-of-care treatments in the context of biofilm-associated CRS. Current guidelines advocate for saline irrigations and topical intranasal corticosteroids (INCS) as first-line therapies to improve mucociliary clearance and reduce inflammation (57). While high-volume devices are superior to sprays in reaching the paranasal sinuses, they primarily function by mechanically clearing mucus and antigens rather than disrupting the biofilm structure. The viscoelastic properties of the EPS matrix provide strong adhesion that often withstands the hydraulic shear forces of standard irrigation (58). Furthermore, drug delivery remains a major hurdle. Kłodzińska et al. highlight that the paranasal sinuses are poorly perfused, non-ventilated cavities, making efficient sinonasal drug targeting notoriously difficult (44). Consequently, even potent topical agents may fail to achieve therapeutic concentrations within the biofilm niche due to anatomical constraints and the viscoelastic properties of the biofilm matrix. Furthermore, while systemic antibiotics are frequently utilized, their long-term use is controversial due to mixed efficacy results and the risk of adverse events (57).A fundamental pharmacokinetic mismatch exists: the Minimum Biofilm Eradication Concentration (MBEC) required to eliminate biofilm-embedded bacteria can be 100 to 1000 times higher than the Minimum Inhibitory Concentration (MIC) for planktonic cells. Such high concentrations are often unachievable in serum without causing systemic toxicity, explaining why long-term antibiotic courses frequently yield mixed efficacy results (16).

Functional endoscopic sinus surgery (FESS) is the primary treatment for CRS patients who do not respond to medical therapy. Prospective studies have shown that FESS can significantly reduce biofilm density three months post-surgery, but a key limitation is that the surgery cannot completely eradicate the biofilm (59),and the residual biofilm becomes a source of disease recurrence.

### Emerging anti-biofilm strategies

Due to the high resistance of biofilms to systemic antibiotics, research has shifted towards developing topical therapeutic agents that can act directly on the sinus mucosa. In this field, rabbit and sheep sinusitis models have served as indispensable, stable, and reproducible preclinical research platforms (27, 60).The novel broad-spectrum antimicrobial agent NVC-422 has demonstrated potent, dose-dependent eradication effects on *Staphylococcus aureus* biofilms in a sheep model, with good safety profiles in experimental animals (61).However, colloidal silver solutions in human clinical trials did not show significant efficacy compared to saline controls, and data comparisons with standard antibiotics remain controversial (62).Furthermore, as a “living antibiotic,” phage cocktail therapy has demonstrated highly efficient, broad-spectrum killing activity against biofilms of clinical S. aureus and *P. aeruginosa* strains isolated from CRS patients. Crucially, the cocktail formulation can effectively overcome the emergence of bacterial resistance, and long-term topical application has been proven safe in animal models, suggesting its potential as a chronic maintenance therapy (63–65). In addition to whole phages, their derived key effector molecules, endolysins, also show great potential. Endolysins are enzymes used by phages to lyse the host bacterial cell wall and have advantages such as high efficiency, strong target specificity, and low propensity to induce resistance, earning them the name “enzybiotics”.However, the clinical translation of phage therapy still faces challenges such as manufacturing standardization, potential immunogenicity, and complex regulatory approval processes (66).While Phase 1 clinical trials (e.g.,AB-SA01) have confirmed safety and preliminary efficacy in humans, large-scale randomized controlled trials are still pending (67).Currently, several immunotherapies are available for CRSwNP, including dupilumab, mepolizumab, omalizumab, and stapokibart. These biologics do not exert direct antimicrobial activity; rather, they may indirectly reduce biofilm persistence by attenuating type 2 inflammation and reshaping the local mucosal environment, although current evidence for microecologic effects remains emerging and partly inconsistent (68, 69).This suggests that the effect of biologics on the microecology may not be universally applicable and could be influenced by various factors, such as the patient’s baseline microbiota composition. Additionally, the EPS of the biofilm is the key physical barrier to its drug resistance, with extracellular DNA (eDNA) being an important structural component (70).DNase I, a matrix-degrading enzyme, can specifically degrade eDNA, thereby disrupting the structural integrity of the biofilm (71).Although DNase I itself has no direct bactericidal activity, its “loosening” effect on the biofilm can significantly enhance the penetration and killing efficacy of traditional antibiotics or antimicrobial peptides, achieving synergistic treatment (72).Targeting the biofilm matrix remains a promising avenue to restore antibiotic susceptibility. N-acetylcysteine (NAC), traditionally used as a mucolytic, has recently demonstrated potent antibiofilm properties. Jotic et al. reported that NAC not only degrades key extracellular matrix components, such as polysaccharides and extracellular DNA, but also functions as a biofilm microenvironment modulator by disrupting the structural integrity of staphylococcal biofilms in CRS patients, thereby enhancing the penetration and efficacy of conventional antibiotics (73). By compromising the viscoelastic properties of the biofilm, agents like NAC may facilitate deeper antibiotic diffusion, offering a readily available therapeutic adjunct for resistant infections. Quorum sensing (QS) is a chemical communication system used by bacteria to coordinate collective behaviors such as biofilm formation (74).QS inhibitors (QSIs) can effectively inhibit biofilm formation and pathogenicity without directly killing the bacteria, thus reducing the selection pressure for resistance. Natural or synthetic QSIs, such as garlic extract and furanone compounds, represent a highly promising “anti-virulence” therapeutic strategy (70).In recent years, novel drug delivery technologies have made significant progress in optimizing the use of existing antibiotics. Among them, nanosystems are considered to have the potential to make important contributions due to their physicochemical properties being similar to those of biofilms. Nanoparticles (NPs) can be direct antimicrobial agents, and they can also serve as effective carriers to deliver antimicrobial agents to the site of infection (75).

### Ecological restoration: nasal microbiota transplantation

Moving beyond eradication, the ultimate goal of CRS management is to restore a resilient, health-associated sinonasal microecology. NMT, inspired by the success of fecal microbiota transplantation, represents a cutting-edge strategy to re-establish this equilibrium. Gill et al. recently reported a case series demonstrating that NMT is a feasible therapeutic option for recalcitrant CRS patients who have failed maximal medical and surgical therapies (76). However, its clinical application is currently limited by significant hurdles, including undefined optimal delivery methods and potential safety risks regarding pathogen translocation. Consequently, it remains an experimental intervention requiring rigorous validation in controlled trials before broader adoption.

To address this, the field can refer to the Preferred Reporting Items for Microbiotherapy (PRIM) guidelines (77). Although PRIM is designed as a universal reporting framework for microbiotherapies across medical disciplines, its rigorous criteria for donor screening and preparation protocols provide an essential reference for standardizing future clinical trials (**Table 3**).

**Table 3:** Clinical translation status of emerging anti-biofilm therapies for CRS.

Therapy category	Specific agent/Example	Current evidence level	Regulatory status	Safety considerations	Availability
Phage Therapy	AB-SA01 (S. aureus cocktail)	Phase 1 Clinical Trial (Ooi et al., 2019 (67)): Safe and well-tolerated; preliminary reduction in bacterial load.	Investigational New Drug (IND)/Compassionate Use only.	Generally safe; low risk of adverse events in early trials. Potential immunogenicity with systemic use.	Experimental/Compassionate Use (Not commercially available for CRS).
Colloidal Silver	Silver Nanoparticles (AgNPs)	RCTs (Scott et al., 2017 (62)): Mixed results. Safe but no significant superiority over saline/antibiotics.	Supplement/Device (varies by region). Not FDA-approved as a CRS drug.	Risk of Argyria (skin discoloration) with long-term excessive use; local irritation.	Commercially Available (often as supplement, off-label use).
Matrix Degrading Agents	Dornase alfa (DNase I)/NAC	Off-label Clinical Use (Mainz et al., 2011 (78); Kłodzińska et al., 2016 (44)); Ex vivo efficacy (Wongkaewkhiaw et al., 2020 (72); Jotic et al., 2025 (73)).	FDA-approved for Cystic Fibrosis (Pulmozyme); Off-label for CRS.	Well-tolerated (in CF); potential for local irritation or pharyngitis.	Available (Off-label prescription).
Quorum Sensing Inhibitors (QSIs)	Furanone derivatives	Preclinical studies show efficacy against biofilms(Shrestha et al., 2022 (70)).	Preclinical.	Toxicity and safety profile unknown in humans.	Experimental.
Immunomodulatory Agents/Biofilm Inhibitors	Azithromycin (Macrolides)	RCTs confirmed immunomodulatory effects in CF and DPB (Shinkai et al., 2008 (79)); Mixed results in CRS (Naclerio & Baroody 2016 (57)).	Approved as an antibiotic. Immunomodulatory use is often off-label.	Macrolides: Cardiac risks (QT prolongation) (Naclerio & Baroody 2016 (57)).	Commercially available.
Nanosystems	Mucus-penetrating particles (MPP)	Ex vivo (Human mucus penetration studies) ((Lai et al., 2011 (80)); Preclinical animal models (Bianchera et al., 2020 (75)).	Preclinical/Early R&D.	Biocompatibility and clearance from the nasal-brain barrier need assessment (Kłodzińska et al., 2016 (44), Bianchera et al., 2020 (75)].	Experimental.
Ecological Restoration	Nasal Microbiota Transplantation (NMT)	Case Series (Gill et al., 2024 (76))Feasible and potential for symptom improvement in recalcitrant cases.	Experimental (Not FDA approved).	Risk of transmitting pathogens; requires rigorous donor screening (applying universal standards like PRIM (77)).	Experimental

## Research challenges and future perspectives

A core theoretical controversy is whether microbial biofilms in CRS are the initiating factor of the disease or merely a secondary phenomenon in the context of a chronic host inflammatory environment. Importantly, although biofilms are frequently detected in CRS, they are not universally present across patients or studies, indicating that they cannot serve as the sole explanation for disease initiation or persistence in all cases. In addition,some studies have detected the presence of biofilms on the sinus mucosa of healthy control groups (49, 51),challenging the notion that the mere presence of biofilms is necessarily pathogenic. The pathophysiology of CRS may not be solely attributable to the formation of specific pathogen biofilms but should be understood within the broader framework of dysbiosis (81).The dysbiosis theory posits that disease arises not only from the acquisition of pathogens but also from the loss of beneficial commensal bacteria. Several studies have shown that, compared to healthy controls, CRS patients have reduced diversity in their sinus microbiota and a lower abundance of some health-associated genera (82, 83).Within this framework, biofilm formation can be seen as an extreme manifestation of dysbiosis in its later stages.

This theoretical framework leads to another key controversy: is the severity of CRS driven by specific high-virulence strains, or by dysregulation of the broader microbial community, including potentially important but still incompletely defined interactions within polymicrobial biofilms? Limited clinical data suggest that polymicrobial biofilms may be associated with greater disease severity than single-species biofilms in some settings. For example, Foreman et al. (84)reported that polymicrobial biofilms were present in a subset of CRS patients and were associated with higher preoperative symptom and radiologic scores; however, postoperative outcomes were not significantly different, and the authors acknowledged important methodological limitations. These findings support further investigation, but do not yet establish polymicrobial biofilms as a universal or fully characterized driver of CRS severity. Other studies have delved into the strain level, finding that S. aureus strains from CRSwNP patients have distinct patterns in their genomes and secretomes, and their biofilms exhibit higher metabolic activity and *in vitro* toxicity (33, 48, 85).Furthermore, the exact role of traditional “commensal bacteria” like Staphylococcus epidermidis in biofilms is still debated, with some research suggesting they may be key builders in some cases (54).These controversies highlight the inadequacy of our understanding of the complexity of the sinonasal microecosystem.

To clarify these complex theoretical debates, more refined research tools are urgently needed. The application of single-cell RNA sequencing (scRNA-seq) technology in recent years represents a major leap forward, revolutionizing our understanding of the host immunopathology of CRS (86). These studies have revealed the unique gene expression profiles of cell subpopulations with unprecedented resolution, identifying highly heterogeneous immune cell infiltration and the key molecular pathways driving different CRS endotypes (87, 88).However, standard scRNA-seq inherently dissociates tissue, causing a loss of crucial spatial information regarding microbial colonization. Previous studies have already demonstrated that the sinonasal microbiome exhibits significant spatial heterogeneity, with distinct bacterial communities occupying specific niches within the sinuses (89). To bridge this gap, future research must integrate scRNA-seq with Spatial Transcriptomics and Dual RNA-seq technologies. Pioneering studies applying techniques like par-seqFISH to bacterial biofilms have already demonstrated the power of this approach, successfully visualizing how distinct physiological states coexist within microns of each other and mapping functional interactions at the microscale (90). Applying such spatial transcriptomics to CRS tissues will allow us to visualize the specific host-pathogen neighborhoods, identifying exactly how localized biofilm aggregates alter the transcriptional state of the adjacent epithelium. This multi-dimensional precision, combining cellular identity with spatial location, will powerfully advance CRS into an era of precision medicine. Beyond mechanistic insight, a major translational priority is the establishment of standardized diagnostic criteria and clinically feasible detection tools for sinonasal biofilms, without which reproducible patient stratification and biofilm-targeted therapeutic trials will remain difficult.

## Conclusion

In summary, existing research has strongly implicated microbial biofilms as a key pathological entity in recalcitrant chronic rhinosinusitis. Their presence is not only closely linked to disease severity and poor prognosis but is also a significant contributor of treatment resistance. In-depth analysis shows that biofilms in CRS are not simple bacterial aggregates but dynamic, fortress-like microbial communities. Their pathogenicity stems from a vicious cycle: the survival strategies of bacteria interact with host susceptibility factors, continuously exacerbating and solidifying the chronic inflammatory state of the sinuses through immune manipulation and intracellular activation pathways. However, the limitations of current diagnostic methods are the primary obstacle to identifying and effectively managing biofilms in current clinical practice.

**Figure 2:**
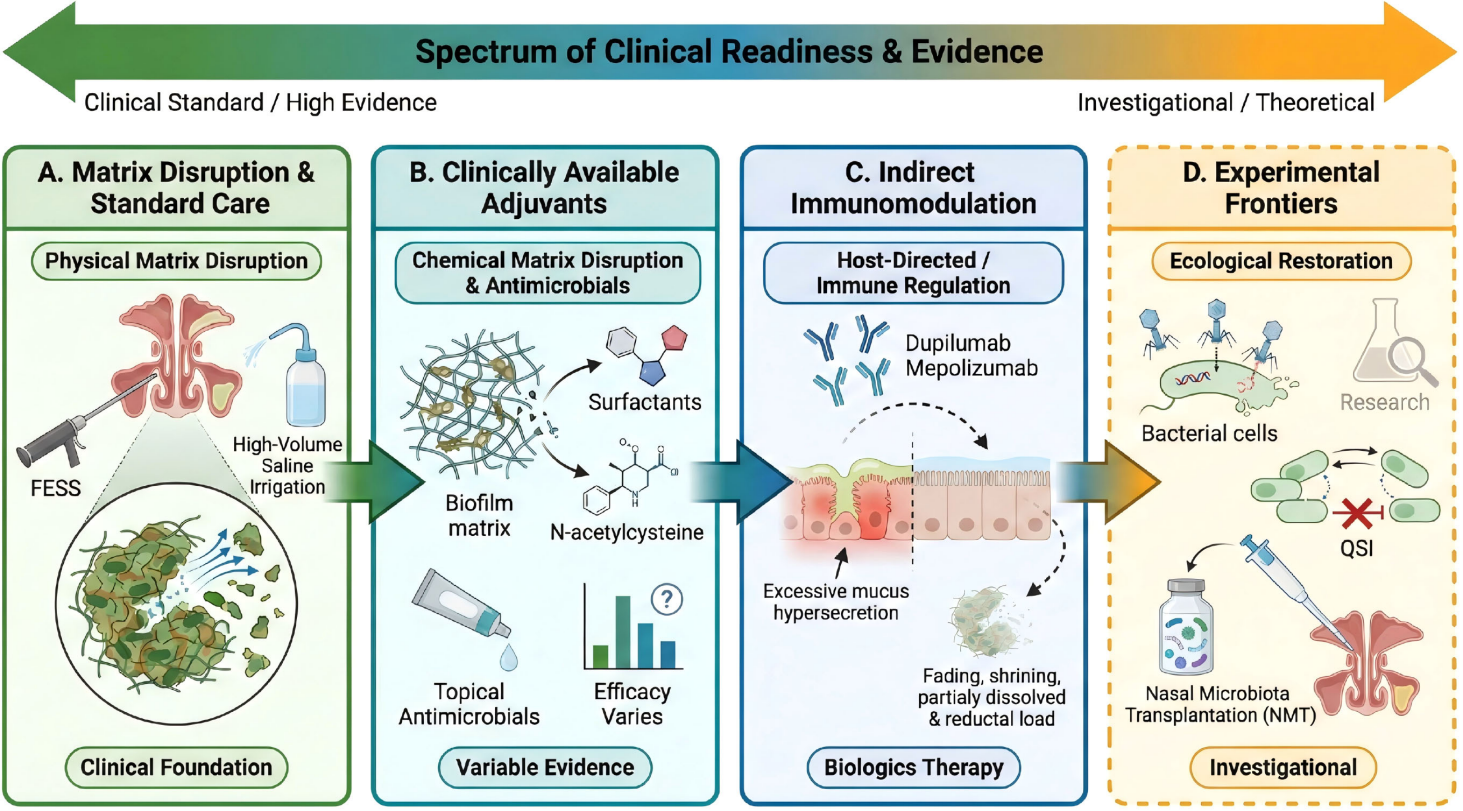
A translational roadmap for biofilm management in CRS. This figure categorizes therapeutic strategies by clinical readiness. **(A)** Standard of Care: Foundation based on physical biofilm disruption via surgery (FESS) and saline irrigation. **(B)** Clinical Adjuvants: Widely available chemical agents (e.g., matrix disruptors, topical antimicrobials) with variable evidence. **(C)** Indirect Immunomodulation: Biologics that target host immune dysregulation to undermine biofilm stability. **(D)** Experimental Frontiers: Investigational ecological approaches (e.g.,Phage therapy, quorum sensing inhibitors, microbiota transplantation) aimed at restoring a healthy microbiome.

Looking ahead, the field is moving towards a new paradigm of multi-dimensional, comprehensive biofilm management. Precision diagnosis will be essential to translating this paradigm into clinically actionable patient stratification and therapy. Current therapeutic strategies for biofilm-associated CRS vary significantly in their level of evidence and clinical availability. To clarify the distinction between established protocols and experimental frontiers, we present a translational roadmap (**Figure 2**). Matrix Disruption & Standard Care: High volume saline irrigation and FESS remain the clinical standard for physical matrix disruption. Adjuvant therapies targeting the biofilm matrix chemically, such as surfactants and N-acetylcysteine, are clinically available but offer variable efficacy. Indirect immunomodulation: Biologics such as dupilumab, mepolizumab, and stapokibart may indirectly affect biofilm persistence by correcting host immune dysregulation and improving the local mucosal environment. Experimental Frontiers: Strategies focusing on ecological restoration such as bacteriophage cocktails and Nasal Microbiota Transplantation (NMT),offer promising theoretical potential but currently lack standardized protocols, confining them to the realm of investigational use.

The most forward-looking research focuses on preventing biofilm formation at its source, for example, by developing small molecule drugs that specifically block key adhesion molecules or interfere with bacterial quorum sensing signal systems.

In conclusion, we are at a crucial research turning point, moving from a traditional bactericidal mindset to a new era of comprehensive management that integrates precision diagnosis, targeted eradication, ecological restoration, and immunomodulation. This paradigm shift from broad-spectrum eradication to ecological restoration offers a theoretically compelling roadmap. While experimental interventions like NMT require further rigorous validation, the integration of precision diagnosis, immunomodulation, and targeted anti-biofilm strategies provides a more comprehensive framework for managing the complex challenge of recalcitrant chronic rhinosinusitis.
